# Quantifying the Impact of Chronic Ischemic Injury on Clinical Outcomes in Acute Stroke With Machine Learning

**DOI:** 10.3389/fneur.2020.00015

**Published:** 2020-01-24

**Authors:** Yee-Haur Mah, Parashkev Nachev, Andrew D. MacKinnon

**Affiliations:** ^1^King's College Hospital NHS Foundation Trust, London, United Kingdom; ^2^School of Biomedical Engineering and Imaging Sciences, King's College London, London, United Kingdom; ^3^High-Dimensional Neurology, Institute of Neurology, University College London, London, United Kingdom; ^4^St George's University Hospitals NHS Foundation Trust, London, United Kingdom

**Keywords:** stroke, prognosis, computed tomography, machine learning, segmentation

## Abstract

Acute stroke is often superimposed on chronic damage from previous cerebrovascular events. This background will inevitably modulate the impact of acute injury on clinical outcomes to an extent that will depend on the precise anatomical pattern of damage. Previous attempts to quantify such modulation have employed only reductive models that ignore anatomical detail. The combination of automated image processing, large-scale data, and machine learning now enables us to quantify the impact of this with high-dimensional multivariate models sensitive to individual variations in the detailed anatomical pattern. We introduce and validate a new automated chronic lesion segmentation routine for use with non-contrast CT brain scans, combining non-parametric outlier-detection score, Zeta, with an unsupervised 3-dimensional maximum-flow, minimum-cut algorithm. The routine was then applied to a dataset of 1,704 stroke patient scans, obtained at their presentation to a hyper-acute stroke unit (St George's Hospital, London, UK), and used to train a support vector machine (SVM) model to predict between low (0–2) and high (3–6) pre-admission and discharge modified Rankin Scale (mRS) scores, quantifying performance by the area under the receiver operating curve (AUROC). In this single center retrospective observational study, our SVM models were able to differentiate between low (0–2) and high (3–6) pre-admission and discharge mRS scores with an AUROC of 0.77 (95% confidence interval of 0.74–0.79), and 0.76 (0.74–0.78), respectively. The chronic lesion segmentation routine achieved a mean (standard deviation) sensitivity, specificity and Dice similarity coefficient of 0.746 (0.069), 0.999 (0.001), and 0.717 (0.091), respectively. We have demonstrated that machine learning models capable of capturing the high-dimensional features of chronic injuries are able to stratify patients—at the time of presentation—by pre-admission and discharge mRS scores. Our fully automated chronic stroke lesion segmentation routine simplifies this process, and utilizes routinely collected CT head scans, thereby facilitating future large-scale studies to develop supportive clinical decision tools.

## Introduction

The functional organization of the brain is highly complex. The clinical consequences of focal brain injury therefore depend not merely on the volume of damaged tissue but also on its anatomical location (1, 2). In stroke, a multiplicity of locations is commonly affected, forming a complex anatomical pattern shaped by the vascular supply to the brain. Optimal prediction of clinical outcomes in stroke then depends on understanding the relation between the patterns of injury and the underlying functional anatomy. This relation is commonly oversimplified, treating most of the resultant variability as noise, with interventional studies usually modeling only the volume of the lesion, or its gross anatomical location, and prognostic studies usually identifying a small number of variables, such as the proportion of the corticospinal tract affected (3–6). The unmodeled variability degrades the sensitivity for detecting interventional effects (7) and limits the predictive power of prognostic measures. Given sufficient data, machine learning-enabled, high-dimensional models drawing on thousands of anatomical variables taken together can capture the underlying complexity, with potentially dramatic impact on inferential and predictive performance (8–10).

These considerations apply not only to acute stroke, but also to the background ischemic damage frequently superimposed on it, with an estimated 10 additional silent infarcts for every symptomatic stroke (11). Such infarcts unsurprisingly have been shown to worsen prognosis (12, 13), confirming the need to model their impact on the acute outcome.

We seek to quantify the predictive value of high-dimensional modeling of background ischemic damage in stroke, and to enable such modeling at large scale, within the current clinical routine.

## Materials and Methods

### Patients

In this single center retrospective observational study, we evaluated all patients presenting to St George's Hospital, London, UK between January 2015 and December 2016, recorded in the local collected Sentinel Stroke National Audit Programme (SSNAP) database, managed within the hyper-acute thrombolysis pathway, and imaged with a computer tomography (CT) head scan on admission. Three hundred and eighty-seven patients who presented with intracerebral hemorrhage were excluded. Also excluded were 403 patients where the images were corrupted by motion or metal artifact or were acquired at external centers. We included all patients with a diagnosis of acute ischemic stroke (*n* = 1,704), and a randomly drawn subset of patients without evidence of acute or chronic stroke on CT (*n* = 78), and another randomly drawn subset of patients with only chronic injury (*n* = 50). This study was approved by the Health Research Authority, Local Research Ethics Committee (London—Camden & King's Cross REC).

### Study Outcomes and Predictors

All patients underwent a plain CT head scan on admission, typically within the first hour of assessment and 3 h of estimated symptoms onset. All imaging was performed on a Siemens SOMATOM definition flash CT scanner and consisted of axially acquired 512 × 512 volumes of typical in-plane resolution of 0.3 × 0.3 mm and a z-plane resolution between 3 and 5 mm.

The following demographic and clinical information were extracted from the routine SSNAP record: age, sex, hypertension, diabetes, congestive heart failure, atrial fibrillation, pre-admission-mRS (pre-mRS), discharge-mRS (dis-mRS), and NIHSS scores. Fifteen patients did not have dis-mRS scores and were excluded from their respective analysis.

Outcome measures were dichotomized to enable the application of classification models. For pre-mRS and dis-mRS, the two categories were low (0–2) vs. high (3–6); for patients who received intravenous (IV) thrombolysis therapy, those with an increase in NIHSS score by more than 2 points after 24 h vs. those whose did not; and patient sex.

### High Dimensional Modeling and Model Evaluation

For each of the 1,704 patients with ischemic stroke, our novel lesion and tissue segmentation and registration routine described below was applied to the admission CT brain scan. This yielded a series of derived volumetric maps projected in standard stereotactic space (Montreal Neurological Institute [MNI]) at a resolution of 4 × 4 × 4 mm^3^. The maps included a binary “lesion mask” where voxels falling within injured tissue are distinguished from all others; an “anomaly map” where voxels are labeled by a statistical measure, zeta (14), of their degree of abnormality; and the gray and white matter tissue probability maps.

We trained a series of SVM models based on LibSVM (15) using the demographic, co-morbidities and neuroimaging data. The models in the series are hierarchically organized to incorporate progressively more information as it naturally becomes available during a patient's admission. We thus first examined models using age only, and incrementally increased the complexity by adding history and examination features followed by neuroimaging. Neuroimaging features included total lesion volume, voxel-level lesion mask, and the combined voxel-level lesion mask and zeta map. Radial basis function (rbf) kernels were used to train the SVM models and were evaluated using a k-fold (*k* = 10) cross-validation technique (16). The optimal parameters were identified using a grid search with the performance for each parameter combination being the area under the receiver operator curve (AUROC) averaged across the 10-folds. Model AUROC values were compared (17), and a bootstrapping technique (*n* = 1,000) was applied to obtain the 95% confidence intervals (CI) for each of the optimal models.

### Background Lesion Segmentation

All CT image pre-processing was performed in SPM12 (18) and in-house developed software written in MATLAB 2016 (19). Pre-processing of the CT image involved affine alignment to the mid-sagittal plane, and a signal intensity transformation using the method described by Rorden et al. (20), to emphasize the tissue-cerebrospinal fluid contrast. SPM12's combined segmentation-normalization routine (21) was then used to generate the transformation parameters to warp the CT image between MNI and the CT scan's native space.

We address the problem of lesion segmentation by first creating 3 binary maps describing regions that are non-lesion (healthy tissue, sulci and ventricles). The image was thresholded at 100 Hounsfield units (HU) to remove bone, while all voxels below zero were clamped to zero, and the image filtered using a Total Variation (TV) algorithm (22) to improve the tissue-CSF contrast. For the healthy tissue map the TV processed image was passed through a top-hat filter. For the sulci map, the TV image was clustered into 3 classes based on signal intensity (14, 24, and 34 HU) using a 3-dimensional Maximum Flow Minimum Cut (23–25) (MFMC) algorithm. The probability map based on the guide signal value of 14 HU was then thresholded. Third, for the ventricular system, the TV image was passed to the MFMC algorithm, but this time clustered into two classes (20, 27 HU). The probability map for the lower guide signal was thresholded to reveal the ventricular system. As the ventricular system exhibits symmetry across the mid-sagittal plane, each voxel was assessed to determine whether its mirror-pair was similarly labeled, and incongruent voxel pairs removed. Finally all three maps were then processed individually with a 2-dimensional watershed transform (26) to cluster similar voxels together.

A map identifying lesioned regions is created from the TV image, by using the MFMC algorithm and specifying a signal range with a higher sensitivity for lesion voxels. From this lesion map, the 3 non-lesion maps are subtracted. Clusters residing along the medial margins of the lateral ventricles, that extend across the mid-sagittal plane were removed. This forms the first binary lesion mask.

By spatially normalizing a set of healthy brains, we can then index each voxel in our test brain according to how different it is from the reference population, thereby creating a map defining abnormal regions based on location and signal value (27). Here we use the zeta anomaly score, using a method detailed and validated in Mah et al. (28) to identify the lesioned regions. A second binary lesion mask is created by subtracting the sulci and ventricle binary maps from the zeta map and thresholding the resultant image.

The two binary lesion masks are combined to form a single binary mask, and then clustered using a 2-dimensional watershed transform created from the CT image. Only watershed regions with a minimum occupancy of 50% are preserved. Finally, a noise reduction step is performed to remove small clusters and very large clusters that only span a single plane. A flow diagram of the process is available in the Supplementary Material.

To validate the accuracy of our automated background lesion segmentation routine, 50 lesion masks of chronic stroke lesions were manually segmented from the axial CT scans in native space with ITK SNAP (29) by a neurologist experienced in the task (YM) and reviewed by an experienced neuroradiologist (ADM). These manual lesion masks represented the “ground truth” for the chronic lesion parameters against which the automated segmentation masks were compared. The performance of the lesion routine was assessed using statistical metrics of sensitivity, specificity, and Dice Similarity Coefficient (30) (DSC), with all statistics derived from images in MNI space.

## Results

The mean (standard deviation, SD) age of the acute ischemic stroke dataset was 73 (15) years, and the 50 patients with only chronic lesions was 78 (49) years. The reference dataset of CT scans without evidence of acute or chronic lesions was significantly younger [mean 49 (16) years] compared against the other two datasets (*p* < 0.001 for both). All comparisons with co-morbidities and sex did not reach statistical significance (Table 1). The relevant patient characteristics and clinical information for the acute ischemic stroke dataset and its subsequent dichotomization are shown in Table 2.

**Table 1:** Population statistics and feature distribution for the three datasets.

	**Acute ischemic stroke**	**No acute or chronic lesion**	**Chronic lesion**
Subjects	1,704	78	50
Age, mean (SD), yr	73 (15)	49 (16)*	73 (12)
Male (%)	853 (50.0)	35 (44.9)	25 (50.0)
Diabetes (%)	470 (27.6)	15 (19.2)	22 (44.0)
Hypertension (%)	1,105 (64.8)	29 (37.2)	39 (78.0)
Atrial fibrillation (%)	422 (24.8)	4 (5.1)	14 (28.0)
Congestive cardiac failure (%)	123 (7.2)	9 (11.5)	6 (12.0)

A total of 1,704 patients were included who had a diagnosis of acute ischemic stroke. A randomly drawn subset of patients without evidence of acute or chronic stroke on CT, and another randomly drawn subset of patients with only chronic injury. There was a significant difference in age between the subset of patients without evidence of acute or chronic stroke on CT, and the other two groups (*); while there was no significant difference between the acute ischemic stroke dataset and the chronic injury dataset. No significant difference was found between any of the other pairings with a Mann-Whitney U-test.

**Table 2:** Population characteristics and feature distribution for the 1,704 patients.

	**Pre-admission mRS (0–2)**	**Pre-admission mRS (3–5)**	**Total**
Subjects	1,372	332	1,704
Age*, mean (SD), yr	71 (15)	83 (10)	
Male (%)	740 (53.9)	113 (34.0)	853
Diabetes (%)	365 (26.6)	105 (31.6)	470
Hypertension (%)	874 (63.7)	231 (69.6)	1,105
Atrial Fibrillation (%)	302 (22.0)	120 (36.1)	422
Congestive Cardiac Failure (%)	83 (6.0)	40 (12.0)	123
Thrombolysis (%)	274 (20.0)	31 (9.3)	305
Door to CT, median (IQR), min	25 (11–119)	23 (11–92.3)	24
Onset to CT, median (IQR), min	150 (90–270)	162 (95–253)	153
Onset to thrombolysis, median (IQR), min	130 (101–183)	128 (100.3–179.5)	130
	**Discharge mRS (0-2)**	**Discharge mRS (3–6)**	
Subjects	761	935	1,696
Age*, mean (SD), yr	68.7 (15)	77.0 (14)	
Male (%)	450 (59.1)	400 (42.8)	850
Diabetes (%)	201 (26.4)	267 (28.6)	468
Hypertension (%)	480 (63.1)	621 (66.4)	1,101
Atrial Fibrillation (%)	126 (16.6)	296 (31.7)	422
Congestive Cardiac Failure (%)	31 (4.1)	91 (9.7)	122
Thrombolysis (%)	135 (17.7)	169 (18.1)	304
Door to CT, median (IQR), min	35 (12–134)	20 (10–93)	24
Onset to CT, median (IQR), min	162 (92.3–228.5)	143 (90–252)	153
Onset to thrombolysis, median (IQR), min	131 (105.5–180)	127 (100–186)	130
	**Increase in NIHSS ≤ 2**	**Increase in NIHSS >2**	
Subjects	281	24	305
Age, mean (SD), yr	72.8 (15)	72·9 (14)	
Male (%)	145 (51.6)	18 (75.0)	163
Diabetes (%)	48 (17.1)	23 (95.8)	71
Hypertension (%)	169 (60.1)	14 (58.3)	183
Atrial Fibrillation (%)	61 (21.7)	5 (20.8)	66
Congestive Cardiac Failure (%)	16 (5.7)	3 (12.5)	19
Thrombolysis	281 (100)	24 (100)	305
Door to CT, median (IQR), min	12 (7–17)	12 (10–17.5)	12
Onset to CT, median (IQR), min	94 (72–131.8)	101 (74–174)	95
Onset to thrombolysis, median (IQR), min	130 (100.5–179)	136 (105–210)	130

There were 8 patients who did not have a discharge modified Rankin Scale score and were excluded from the respective analyses. For each prediction model (pre-admission modified Rankin Scale score, discharge modified Rankin Scale score, and deterioration in National Institute of Health Stroke Score of more than 2 points) the data has been arranged according to the corresponding binary groups. Apart from age (*) in the pre-admission and discharge mRS score analyses, no significant difference was found between pairings for the remaining features. For each of the pairings the median (IQR) door to CT, onset to CT and onset to thrombolysis times are presented. No significant difference was found between any of the parings with a Mann-Whitney U-test. For the respective timing measures, the overall median time is shown in the “Total” column.

No significant difference was found between the dichotomized groups except for age, where patients were older in the high pre-mRS and dis-mRS groups. The prevalence of identified background injury was 1,520/1,704, in the context of a distribution pre-mRS scores consistent with previous studies (31).

**Figure 1:**
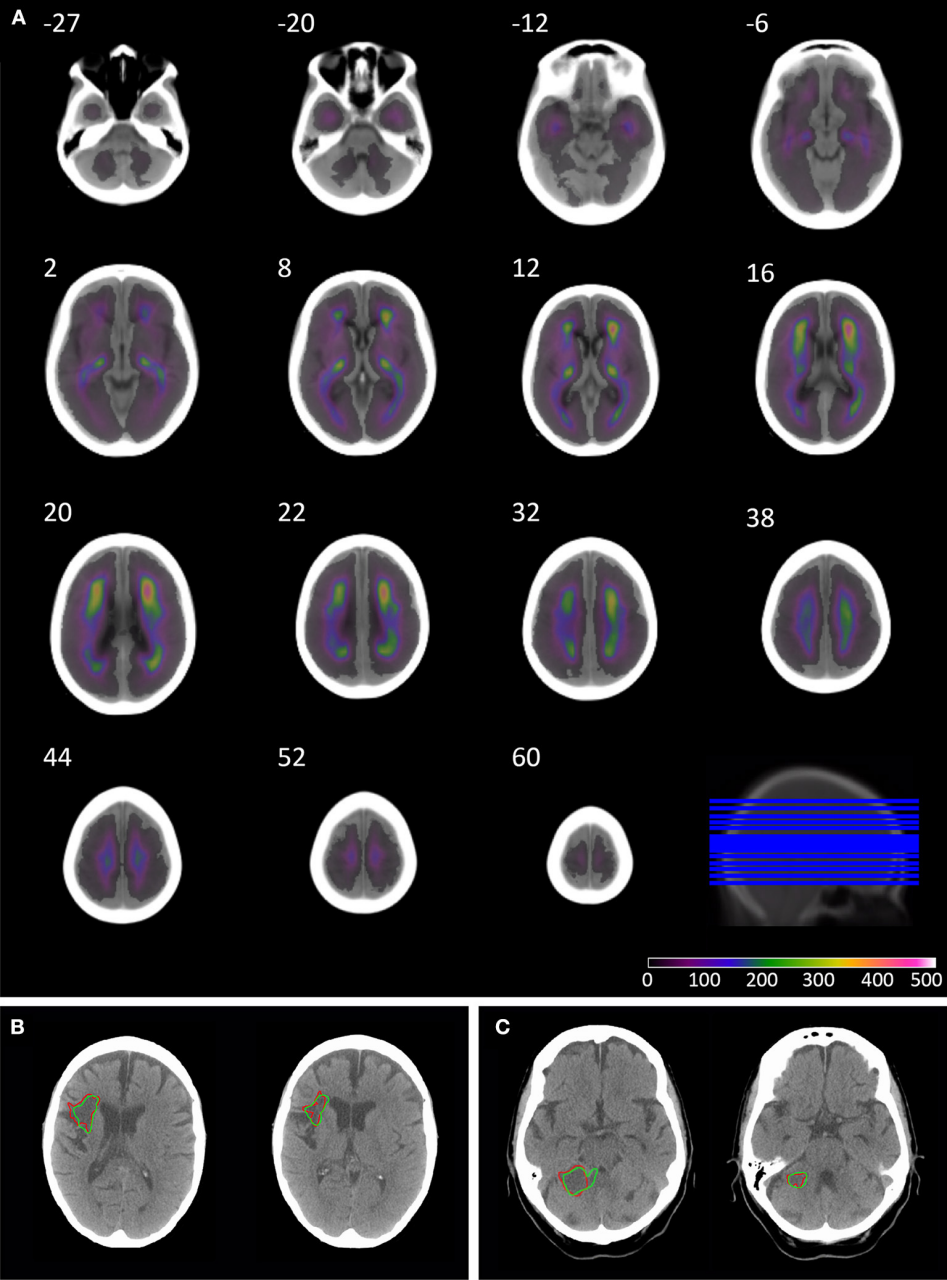
Overlay coverage of the 1520 lesion segmented CT brain scans, and example automated segmentation results. The overlay shows the coverage of the 1520 lesions segmented using the automated routine **(A)**. The underlay image is the mean image created from the subset of 78 patients without evidence of acute or chronic stroke on CT, sliced axially at the following co-ordinates: −27, −20, −12, −6, 2, 8, 12, 16, 20, 22, 32, 38, 44, 52, and 60. Axial slices through patients' 3 **(B)** and 49 **(C)** CT brain scan in native space. The borders of the lesion are depicted in red for manual segmentation and green for the automated segmentation routine.

The anatomical pattern of injury across the population shows the highest density in the anterior and posterior watershed territories of the middle cerebral artery, and posterior thalamus, with lower densities around the cerebellum and posterior fossa (Figure 1).

### Pre-admission mRS: 0–2 vs. 3–5

**Figure 2:**
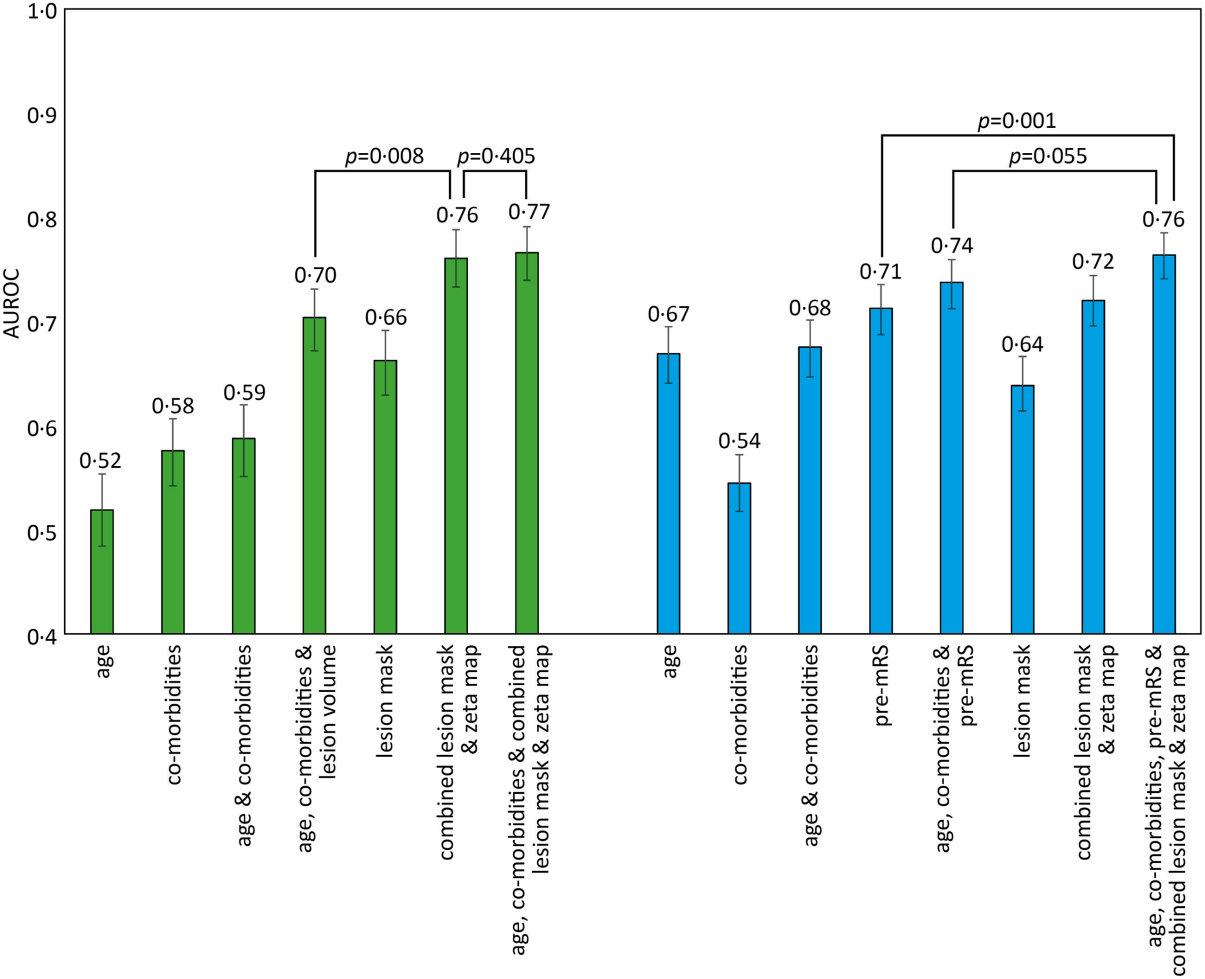
AUROC results for different SVM models trained to predict pre-admission and discharge mRS scores. The bar chart shows the Area Under the Receiver Operator Curve (AUROC) values obtained for different support vector machine (SVM) models using a radial basis function (rbf) kernel. The comorbidities included in the models were diabetes, hypertension, atrial fibrillation, and congestive cardiac failure. Pre-admission mRS scores 0–2 vs. 3–5 (green), and discharge mRS score 0–2 vs. 3–6 (blue), with 95% confidence intervals shown as errors bars. Pairwise AUROC comparisons are shown with brackets and *p*-values adjacent.

Classification models trained to discriminate between the low and high pre-mRS groups showed increasing performance with the incremental addition of clinical and imaging features (Figure 2).

**Figure 3:**
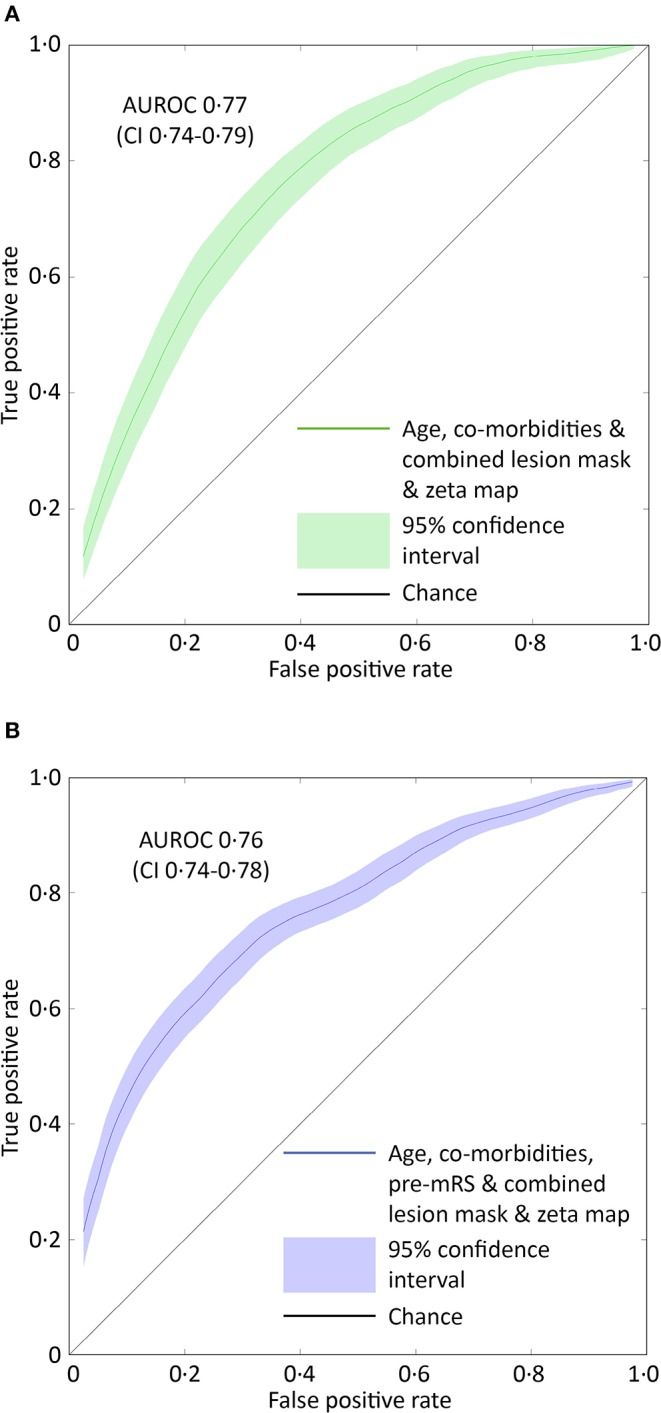
ROC curves for the optimal SVM model predicting pre-admission and discharge mRS scores. **(A)** The receiver operator characteristics curve for the best performing support vector machine model (combined lesion mask and zeta map) trained to classify patients as either 0–2 or 3–5 based on the pre-admission mRS score. The Area Under the Receiver Operator Curve (curved line) is 0.77 (95% CI 0.74–0.79, green shaded area). **(B)** The receiver operator characteristics curve for the best performing support vector machine model (combined lesion mask and zeta map with pre-admission modified Rankin Scale score) trained to classify patients as either 0–2 or 3–6 based on the discharge-mRS score. The Area Under the Receiver Operator Curve (curved line) is 0.76 (95% CI 0.74–0.78, blue shaded area). The straight black line represents a predictive performance of pure chance.

Models based on clinical features alone performed worst, exhibiting an AUROC of <0.60. The addition of lesion volume to clinical features increased this to 0.70 (95% CI 0.67–0.73). In comparison, the combined lesion mask and zeta map alone was significantly different at 0.76 (95% CI 0.73–0.79, *p* = 0.008). The addition of clinical features did improve the AUROC to 0·77 (95% CI 0.74–0.79), whose receiver operating curve is shown in Figure 3A, but the improvement did not reach significance (*p* = 0.405).

### Discharge-mRS Score: 0–2 vs. 3–6

Classification models trained to discriminate between the low and high dis-mRS score groups showed a similar pattern of performance with increasing clinical features. The model using the pre-mRS score alone achieved an AUROC of 0.71 (95% CI 0.69–0.74) which was not significantly different to the model using the combined lesion mask and zeta map (AUROC 0.72, 95% CI 0.70–0.74, *p* = 0.340). The optimal model incorporated the clinical features, pre-mRS and imaging (in the form of the combined lesion mask and zeta map) achieving an AUROC of 0.76 (95% CI 0.74–0.78) (Figure 3B). However, it did not perform significantly better than the same model with imaging information excluded (AUROC 0.74, 95% CI 0.71–0.76, *p* = 0.055).

### Increase in NIHSS Score of More Than 2 Points Following Thrombolytic Therapy

**Figure 4:**
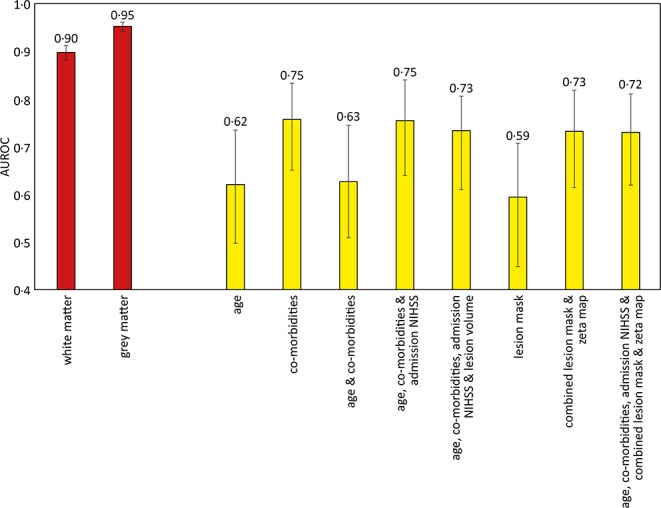
Area under the receiver operator curve results for different support vector machine models trained to predict sex and deterioration in NIHSS score. The sex discrimination models (red) were trained using linear kernels, while the increase of more than 2 points in the NIHSS score (yellow) used radial basis function kernels. 95% confidence intervals shown as error bars. The comorbidities included in the models were diabetes, hypertension, atrial fibrillation, and congestive cardiac failure.

There were 305 patients who received IV thrombolysis therapy with available admission and 24-h post thrombolysis NIHSS scores recorded. Increments in modeled clinical features (age, co-morbidities, and admission NIHSS score) accompanied an increase in AUROC, with the optimal model using all these features (AUROC 0.75, CI 0.64–0.84). The addition of imaging information did not result in a significant difference in predicting future patient deterioration (Figure 4).

### Sex Prediction

The gray and white matter probability maps for the 1,704 patients were extracted from the normalized plain CT scans and used to train an SVM model to determine the sex of the patient, as an internal quality control of the image segmentation process and subsequent modeling. The linear kernel model using gray matter maps performed the best, achieving an AUROC of 0.95 (95% CI 0.94–0.96), shown in Figure 4.

### Automated Chronic Lesion Segmentation Evaluation

**Figure 5:**
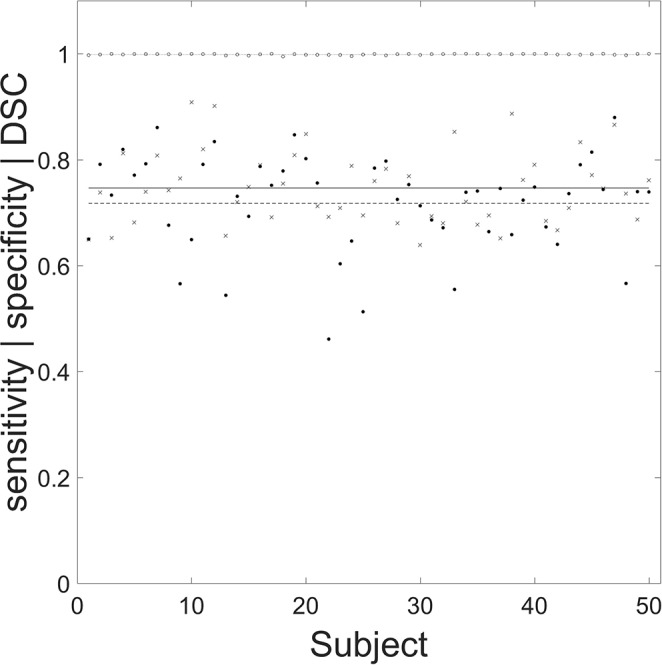
Performance of the automated segmentation routine for the 50 chronic stroke lesions. Filled circles, crosses, and open circles correspond to specificity, sensitivity, and Dice similarity coefficient (DSC), respectively. The mean (SD) values for specificity (dashed line), sensitivity (solid line), and DSC (dotted line) are 0.746 (0.069), 0.999 (0.001), and 0.717 (0.091), respectively. The mean (SD) age for the 50 manually segmented chronic lesions was 73 (12) years, with 25 male patients.

The mean (SD) sensitivity, specificity and DSC of the segmentation routine were 0.746 (0.069), 0.999 (0.001), and 0.717 (0.091), respectively. The individual performance statistics are shown (Figure 5).

## Discussion

With the digitization of neuroimaging and its extensive use in stroke medicine, there is an opportunity to capitalize on recent advances in machine learning to develop predictive models of sufficient individual-level accuracy to support clinical decisions. We have shown that complex, high-dimensional models of brain injury have better predictive power compared with simple, low-dimensional ones incorporating only age and lesion volume. Our fully automated chronic lesion segmentation routine simplifies the necessary image pre-processing, facilitating the interrogation of the large datasets high-dimensional modeling requires.

We have shown that incremental additions of clinical and imaging features improve the performance of predictive models estimating a patient's pre-mRS and dis-mRS scores. Although the high mRS groups for both pre-mRS and dis-mRS analyses were significantly older (*p* < 0.001), the models using age alone, or in combination with co-morbidities failed to exceed an AUROC of 0.68. This result is likely due to the variability in functional level for a specific age, with co-morbidities and age probably acting synergistically as a surrogate marker of the general health of the patient.

Past studies have demonstrated that modeling the acute lesion can predict a patient's future functional ability, therefore intuitively, modeling chronic lesions should provide an insight into a patient's pre-admission level of function, which is reflected in our results. We also found that high-dimensional neuroimaging models that use each voxel as a separate dimension achieved better AUROC values, suggesting that there is an additional benefit in modeling the pattern of damage. Focal injuries are believed to influence the structure of the brain distant to the original lesion, with diaschisis playing a critical role in stroke recovery (32, 33). This phenomenon would be consistent with the observed improvement in performance following the addition of the zeta map to the lesion mask, with a significant increase in AUROC (0.66 vs. 0.76 *p* < 0.0001) as the zeta map encodes changes in the brain distant to the chronic lesion, such as atrophy. The SSNAP database from which the co-morbidity features were retrieved may not reflect the information available at presentation but instead accrued over the patient's admission. This will improve accuracy and minimizes missing data but may positively bias the observed predictive performance of the model using the clinical features to estimate pre-mRS scores. Nevertheless, the addition of clinical features to the combined lesion mask and zeta map did not significantly increase the AUROC.

In the dis-mRS prediction models, the combined lesion mask and zeta map model achieved a similar performance as the model using pre-mRS scores. The optimal dis-mRS model combined all clinical information and neuroimaging into a single model. This was significantly better than using the pre-mRS scores alone, however failed to reach significance when compared against the model using all clinical information (*p* = 0.055).

Early prognostication is a difficult task, especially when information is reliant on human recall or patient interaction, in the hyperacute phase of admission. The THRIVE score (34) used age, stroke severity (as determined with the NIHSS score) and comorbidities (hypertension, diabetes and atrial fibrillation), to estimate the likelihood of an mRS score of <3 at 90 days. When applied to patients who received endovascular treatment, their AUROC was 0.71, and 0.29 in those who received IV thrombolysis therapy (35). These results are comparable to our model using age and co-morbidities (AUROC 0.68), however their analysis did not include a significant proportion of patients who did not receive recanalization therapy, limiting the clinical application of the THRIVE score. In comparison the ASTRAL score (36) is an integer-based method to estimate the 90-day level of function based on a dichotomized mRS score of 0–2 vs. 3–6, in the emergency room. It managed an impressive AUROC of 0.90, and 0.79 when externally validated on the VISTA cohort (37). The ASTRAL score predominantly used information pertaining to the acute injury, and included an assessment of the visual fields, which can be very challenging in an aphasic or somnolent patient. In contrast our models only used information from the past, with the neuroimaging focusing on the pattern of chronic injury, to estimate the functional independence at discharge rather than at 30 days. By using the information contained within the admission CT head scan, our model achieved an AUROC of 0.72, and thereby minimizes the reliance on operator and patient co-operation. Future work incorporating both the pattern of chronic and acute injury, may improve the performance further.

Our models demonstrate the potential of applying machine learning to neuroimaging to produce clinical tools of value in the clinical management of stroke patients. At the hyper-acute stage, one study has examined the development of patient selection tools to help identify suitable candidates for IV thrombolysis (38) and endovascular clot retrieval (39). The pre-mRS score was identified as an independent predictor of outcome at 90 days, as was a previous history of stroke. Though the interaction with treatment was small, a patient's history of stroke was treated as a binary measure, ignoring the burden of chronic damage, still less its anatomical pattern. Clearly, different patterns of injury will have different functional consequences; a reductive approach ignores valuable information which may shed light on the general health of the patient's brain and its ability to recover from the acute insult.

Sex determination was used as an internal quality control technique for both the image segmentation and subsequent modeling. Comparable work based on MR volumetric imaging has shown sex differences in gray and white matter patterns (40), and are able to differentiate the sex of the subject with an accuracy of 89% (41). Our models based on the gray and white matter probability maps derived from CT scans performed similarly, with an AUROC of 0.95 and 0.90, respectively, and suggests our technique preserves the individual's tissue class differences present in the non-contrast CT scan.

Although this study has shown parameterizing the complex pattern of chronic injury improves our ability to predict the level of functional independence in patients, our work presents some limitations. First, the optimal model to predict a deterioration in NIHSS score after IV thrombolysis therapy achieved an AUROC of 0.75 (95% CI 0.64–0.84). However, unlike the mRS models, increases in model complexity did not demonstrate a significant improvement in performance. The unfavorable ratio of number of patients to number of modeled features means these models may have struggled to capture the high-dimensional signals in the data and would be expected to perform better with a larger dataset. Second, the lesion segmentation routine has been designed to extract the pattern of chronic injury, there may be details relating to the acute lesion in the imaging, as the zeta map is a whole brain representation of anomaly, thus subtle reductions in CT density from the acute lesion may be conveyed numerically to the SVM algorithm. However, the median delay from symptom onset to CT scan time was <3 h, therefore features of the acute lesion are not expected to be readily visible on a plain CT scan. Although we can presume information relating to the acute lesion to be small, we cannot exclude it entirely. Nevertheless, this result suggests the presence of chronic lesions interacts with the presenting acute lesion, and the pattern of injury confers meaningful information for predicting a patient's future course. Third, this retrospective study uses information routinely gathered during clinical practice over 2 years and collected as part of the SSNAP initiative in the UK. The modified Rankin Scale score is a general summary measure of patients' function, which is subject to moderate variability, particularly in the clinical setting (31, 42). Although converting the score to a binary outcome may obviate some of this variability, both aspects will impact on our model's potential to accurately classify patients. Fourth, endovascular clot retrieval (thrombectomy) is becoming more prevalent in routine clinical practice either alone or in combination with thrombolysis. While thrombectomy is the preferred hyperacute intervention, it will only be possible where the thrombus is of sufficient size to be accessed and desirable where thrombolysis is not comparably effective. Currently, the proportion of eligible patients is estimated to be 5–9% (43) and projected to increase to around 22–25% (44). Therefore, the prospect of thrombolysis being wholly superseded by thrombectomy is unlikely, with three quarters of patients with acute ischemic stroke continuing to receive either thrombolysis or no hyperacute treatment. Finally, our study was a single center retrospective observational project, exploring the impact of parameterizing the pattern of chronic injury. Further validation studies are required to assess the generalizability of the models before formal introduction into clinical practice.

Our proposed method facilitates the analysis of large datasets that can power the development of high-dimensional models to address this issue with greater individual-level precision. Our method of combining neuroimaging information can be adapted to incorporate specialized sequences, such as intracranial CT angiography or MR imaging. The substantial logistical difficulties of sufficiently rapid MR, especially in patients with abnormal consciousness or potential contraindications, mean that CT will remain the first line modality of choice in most stroke units for the foreseeable future. Were both CT and MR are performed, the former to guide initial management and the latter for subsequent decision-making, it is possible to combine information from both scans, maximizing the intelligence drawn from the available data. Regardless of the modality, the clinical application of the quantified extent of damage will be to capture additional variability in clinical outcome parameters of any kind for prognostic or prescriptive purpose. Our results further open the possibility of reducing clinical reliance on the patient's recall of his or her history, always a potential problem where dysfunction of any part of the brain may exist.

The detailed characteristics of a stroke patient's CT brain scan contain information about the patient's functional status and risk of deterioration. We have demonstrated that by increasing the number of features used to parameterize the spatial patterns of brain injury on CT, we are able to stratify patients—at the time of presentation—by pre-admission and discharge mRS scores, and to estimate who are likely to have further deterioration in their NIHSS score following IV thrombolysis treatment. Our image segmentation routine exhibits excellent agreement with manual segmentation, and extracts this information in an automated fashion, thereby placing minimal demands on the operator. Our approach enables processing of routinely collected CT scans for training high dimensional models that can support clinical and service decision making, especially during a time-critical and challenging period of the patient's admission.

## Data Availability Statement

The datasets generated for this study will not be made publicly available. The datasets generated for this study are subject to ethical clearance and request to the corresponding author.

## Ethics Statement

The studies involving human participants were reviewed and approved by Health Research Authority, Local Research Ethics Committee. Written informed consent for participation was not required for this study in accordance with the national legislation and the institutional requirements.

## Author Contributions

Y-HM and PN contributed to the conception and design of the study. Y-HM and AM contributed to the acquisition and analysis of the data. All authors contributed to the drafting or critical revision of the manuscript for important intellectual content.

### Conflict of Interest

The authors declare that the research was conducted in the absence of any commercial or financial relationships that could be construed as a potential conflict of interest.

## References

[1] Bang OY Lee PH Heo KG Joo US Yoon SR Kim SY. Specific DWI lesion patterns predict prognosis after acute ischaemic stroke within the MCA territory. J Neurol Neurosurg Psychiatr. (2005) 76:1222–8. 10.1136/jnnp.2004.059998 16107355 PMC1739781

[2] Nazzal ME Saadah MA Saadah LM Trebinjac SM. Acute ischemic stroke: relationship of brain lesion location & functional outcome. Disabil Rehabil. (2009) 31:1501–6. 10.1080/09638280802627702 19479508

[3] Stone SP Allder SJ Gladman JR. Predicting outcome in acute stroke. Br Med Bull. (2000) 56:486–94. 10.1258/0007142001903139 11092097

[4] Schiemanck SK Kwakkel G Post MW Kappelle LJ Prevo AJ. Predicting long-term independency in activities of daily living after middle cerebral artery stroke: does information from MRI have added predictive value compared with clinical information? Stroke. (2006) 37:1050–4. 10.1161/01.STR.0000206462.09410.6f 16497980

[5] Zhu LL Lindenberg R Alexander MP Schlaug G. Lesion load of the corticospinal tract predicts motor impairment in chronic. Stroke. (2010) 41:910–5. 10.1161/STROKEAHA.109.577023 20378864 PMC2886713

[6] Feng W Wang J Chhatbar PY Doughty C Landsittel D Lioutas V-A. Corticospinal tract lesion load: an imaging biomarker for stroke motor outcomes. Ann Neurol. (2015) 78:860–70. 10.1002/ana.24510 26289123 PMC4715758

[7] Xu T Jager HR Husain M Rees G Nachev P. High-dimensional therapeutic inference in the focally damaged human brain. In: Society for Neuroscience Annual Meeting Abstracts. San Diego, CA (2016). 29149245

[8] Chen R Hillis AE Pawlak M Herskovits EH. Voxelwise bayesian lesion-deficit analysis. Neuroimage. (2008) 40:1633–42. 10.1016/j.neuroimage.2008.01.014 18328733 PMC2394734

[9] Smith DV Clithero JA Rorden C Karnath H-O. Decoding the anatomical network of spatial attention. Proc Natl Acad Sci USA. (2013) 110:1518–23. 10.1073/pnas.1210126110 23300283 PMC3557038

[10] Rondina JM Park C Ward NS. Brain regions important for recovery after severe post-stroke upper limb paresis. J Neurol Neurosurg Psychiatr. (2017) 88:737–43. 10.1136/jnnp-2016-315030 28642286 PMC5561379

[11] Leary MC Saver JL. Annual incidence of first silent stroke in the United States: a preliminary estimate. Cerebrovasc Dis Basel Switz. (2003) 16:280–5. 10.1159/000071128 12865617

[12] de Jong G Kessels F Lodder J. Two types of lacunar infarcts: further arguments from a study on prognosis. Stroke. (2002) 33:2072–6. 10.1161/01.STR.0000022807.06923.A3 12154265

[13] Putaala J Haapaniemi E Kurkinen M Salonen O Kaste M Tatlisumak T. Silent brain infarcts, leukoaraiosis, and long-term prognosis in young ischemic stroke patients. Neurology. (2011) 76:1742–9. 10.1212/WNL.0b013e31821a44ad 21576692

[14] Rieck K Laskov P Language models for detection of unknown attacks in network traffic. J Comput Virol. (2007) 2:243–56. 10.1007/s11416-006-0030-0

[15] Chang C-C Lin C-J LIBSVM: a library for support vector machines. ACM Trans Intell Syst Technol. (2011) 3:1–27. 10.1145/1961189.1961199

[16] Kohavi R A study of cross-validation and bootstrap for accuracy estimation and model selection. In Proceedings of the 14th International Joint Conference on Artificial Intelligence–Vol. 2 San Francisco, CA: Morgan Kaufmann Publishers Inc (1995). p. 1137–43.

[17] Hanley JA McNeil BJ. The meaning and use of the area under a receiver operating characteristic (ROC) curve. Radiology. (1982) 143:29–36. 10.1148/radiology.143.1.7063747 7063747

[18] The FiL Methods group (2000). SPM. The FiL Methods Group. Available online at: http://www.fil.ion.ucl.ac.uk/spm/

[19] MATLAB (2000). Matlab. Natick, MA: The MathsWorks Inc.

[20] Rorden C Bonilha L Fridriksson J Bender B Karnath H-O. Age-specific CT and MRI templates for spatial normalization. Neuroimage. (2012) 61:957–65. 10.1016/j.neuroimage.2012.03.020 22440645 PMC3376197

[21] Ashburner J Friston KJ. Unified segmentation. Neuroimage. (2005) 26:839–51. 10.1016/j.neuroimage.2005.02.018 15955494

[22] Strong D Chan T Edge-preserving and scale-dependent properties of total variation regularization. Inverse Probl. (2003) 19:S165–87. 10.1088/0266-5611/19/6/059

[23] Yuan J Schörr C Steidl G Simultaneous higher-order optical flow estimation and decomposition. SIAM J Sci Comput. (2007) 29:2283–304. 10.1137/060660709

[24] Yuan J Bae E Tai X-C A study on continuous max-flow and min-cut approaches. In Proceedings/CVPR IEEE Computer Society Conference on Computer Vision and Pattern Recognition. San Francisco, CA (2010). p. 2217–24. 10.1109/CVPR.2010.5539903

[25] Hutchison D Kanade T Kittler J Kleinberg JM Mattern F Mitchell JC A continuous max-flow approach to potts model. In: Daniilidis K Maragos P Paragios N editors. Computer Vision–ECCV 2010. Berlin; Heidelberg: Springer (2010). p. 379–92.

[26] Meyer F Topographic distance and watershed lines. Signal Process. (1994) 38:113–25. 10.1016/0165-1684(94)90060-4

[27] Gillebert CR Humphreys GW Mantini D. Automated delineation of stroke lesions using brain CT images. Neuroimage Clin. (2014) 4:540–8. 10.1016/j.nicl.2014.03.009 24818079 PMC3984449

[28] Mah Y-H Jager R Kennard C Husain M Nachev P. A new method for automated high-dimensional lesion segmentation evaluated in vascular injury and applied to the human occipital lobe. Cortex. (2012) 56:51–63. 10.1016/j.cortex.2012.12.008 23347558 PMC4071441

[29] Yushkevich PA Piven J Hazlett HC Smith RG Ho S Gee JC. User-guided 3D active contour segmentation of anatomical structures: Significantly improved efficiency and reliability. Neuroimage. (2006) 31:1116–28. 10.1016/j.neuroimage.2006.01.015 16545965

[30] Dice LR Measures of the amount of ecologic association between species. Ecology. (1945) 26:297–302. 10.2307/1932409

[31] Quinn TJ Taylor-Rowan M Coyte A Clark AB Musgrave SD Metcalf AK. (2017). Pre-Stroke modified rankin scale: evaluation of validity, prognostic accuracy, and association with treatment. Front Neurol. 8:275. 10.3389/fneur.2017.00275 28659859 PMC5468801

[32] Seitz RJ Azari NP Knorr U Binkofski F Herzog H Freund HJ. The role of diaschisis in stroke recovery. Stroke. (1999) 30:1844–50. 10.1161/01.STR.30.9.1844 10471434

[33] Price CJ Warburton EA Moore CJ Frackowiak RS Friston KJ. Dynamic diaschisis: anatomically remote and context-sensitive human brain lesions. J Cogn Neurosci. (2001) 13:419–29. 10.1162/08989290152001853 11388916

[34] Flint AC Cullen SP Faigeles BS Rao VA. Predicting long-term outcome after endovascular stroke treatment: the totaled health risks in vascular events score. Am J Neuroradiol. (2010) 31:1192–6. 10.3174/ajnr.A2050 20223889 PMC7965456

[35] Kamel H Patel N Rao VA Cullen SP Faigeles BS Smith WS. The totaled health risks in vascular events (thrive) score predicts ischemic stroke outcomes independent of thrombolytic therapy in the NINDS tPA trial. J Stroke Cerebrovasc Dis. (2013) 22:1111–6. 10.1016/j.jstrokecerebrovasdis.2012.08.017 23122722

[36] Ntaios G Faouzi M Ferrari J Lang W Vemmos K Michel P. An integer-based score to predict functional outcome in acute ischemic stroke: the ASTRAL score. Neurology. (2012) 78:1916–22. 10.1212/WNL.0b013e318259e221 22649218

[37] Quinn TJ Singh S Lees KR Bath PM Myint PK. Validating and comparing stroke prognosis scales. Neurology. (2017) 89:997–1002. 10.1212/WNL.0000000000004332 28794250

[38] Foell RBT Silver B Merino JG Wong EH Demaerschalk BM Poncha F. Effects of thrombolysis for acute stroke in patients with pre-existing disability. Can Med Assoc J. (2003) 169:193–7. 12900476 PMC167119

[39] Venema E Mulder MJHL Roozenbeek B Broderick JP Yeatts SD Khatri P. (2017). Selection of patients for intra-arterial treatment for acute ischaemic stroke: development and validation of a clinical decision tool in two randomised trials. BMJ. 357:j1710. 10.1136/bmj.j1710 28468840 PMC5418887

[40] Good CD Johnsrude I Ashburner J Henson RN Friston KJ Frackowiak RS. Cerebral asymmetry and the effects of sex and handedness on brain structure: a voxel-based morphometric analysis of 465 normal adult human brains. Neuroimage. (2001) 14:685–700. 10.1006/nimg.2001.0857 11506541

[41] Nieuwenhuis M Schnack HG van Haren NE Lappin J Morgan C Reinders AA. Multi-center MRI prediction models: predicting sex and illness course in first episode psychosis patients. Neuroimage. (2017) 145:246–53. 10.1016/j.neuroimage.2016.07.027 27421184 PMC5193177

[42] Fearon P McArthur KS Garrity K Graham LJ McGroarty G Vincent S. Prestroke modified rankin stroke scale has moderate interobserver reliability and validity in an acute stroke setting. Stroke. (2012) 43:3184–8. 10.1161/STROKEAHA.112.670422 23150650

[43] Wiacek M Kaczorowski R Sieczkowski B Kanas N Bartosik-Psujek H. Mechanical thrombectomy: determining the proportion of eligible acute ischemic stroke patients in the cohort of single academic stroke center. Neurol Neurochir Pol. (2018) 52:359–63. 10.1016/j.pjnns.2017.12.010 29331206

[44] Mokin M Ansari SA McTaggart RA Bulsara KR Goyal M Chen M. Indications for thrombectomy in acute ischemic stroke from emergent large vessel occlusion (ELVO): report of the SNIS Standards and Guidelines Committee. J Neurointerventional Surg. (2019) 11:215–20. 10.1136/neurintsurg-2018-014640 30610069

