# Supplementary material for: Quantifying the Impact of Chronic Ischemic Injury on Clinical Outcomes in Acute Stroke With Machine Learning
Source: Front Neurol. 2020 Jan 24;11:15. doi: 10.3389/fneur.2020.00015 (PMC6992664; doi:10.3389/fneur.2020.00015)
Supplement: Supplementary file 1 [file Data_Sheet_1.PDF]

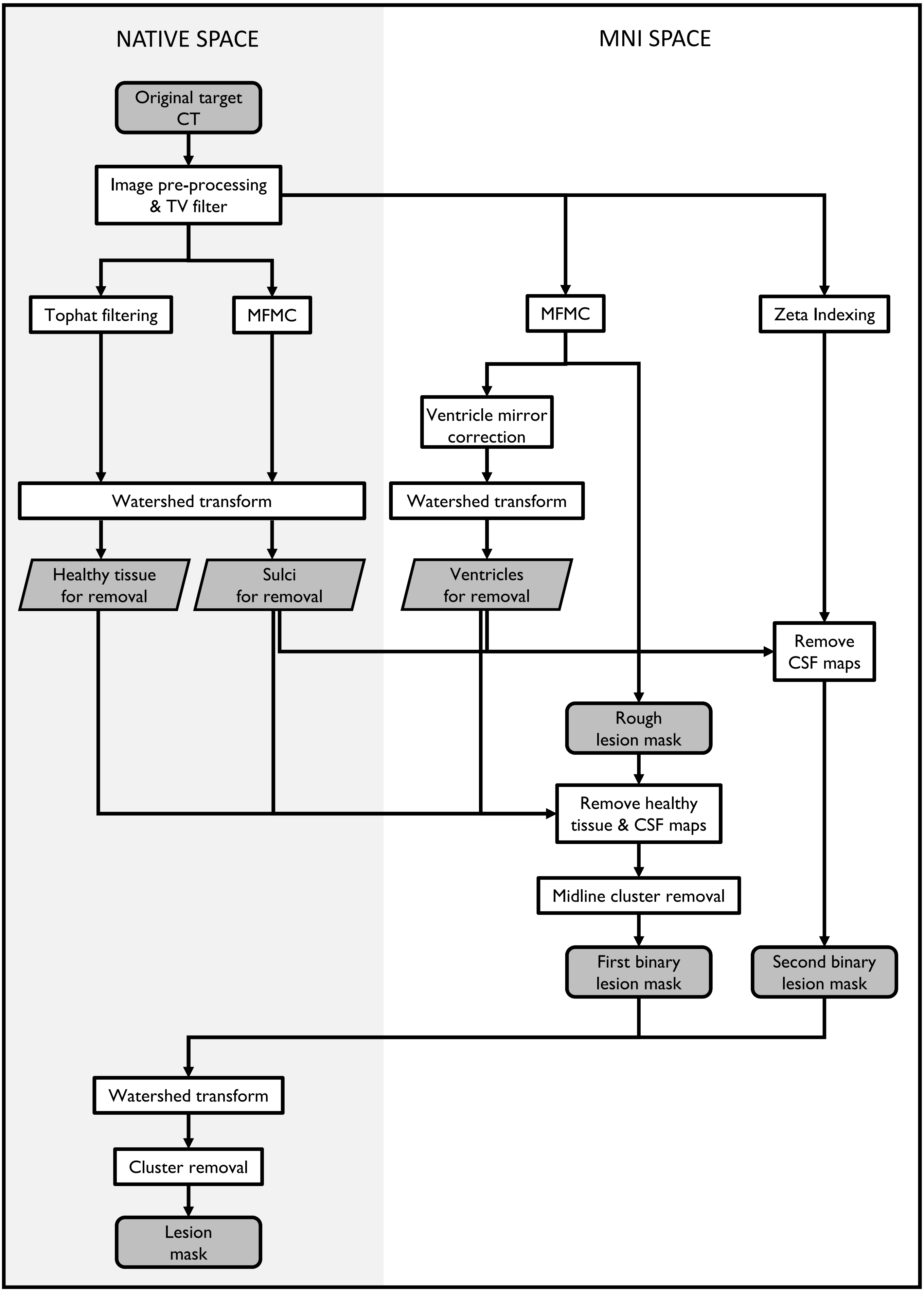

Flow diagram showing the steps to create the probability maps and final binary lesion mask. The different volumes were warped between the CT's native space and MNI space, using the transformation parameters obtained via SPM12's combined segmentation-normalisation routine. Total Variation (TV) filer; Maximum flow minimum cut (MFMC);
